# What is the Proximal Cause of Aging?

**DOI:** 10.3389/fgene.2012.00189

**Published:** 2012-09-25

**Authors:** Piotr Zimniak

**Affiliations:** ^1^Department of Pharmacology and Toxicology, University of Arkansas for Medical SciencesLittle Rock, AR, USA; ^2^Central Arkansas Veterans Healthcare SystemLittle Rock, AR, USA

In spite of exciting new insights into regulatory mechanisms that modulate the aging process, the proximal cause of aging remains one of the unsolved big problems in biology. An evolutionary analysis of aging provides a helpful theoretical framework by establishing boundary conditions on possible mechanisms of aging. The fundamental insight is that the force of natural selection diminishes with age (Medawar, 1952; Comfort, 1956; Williams, 1957). This does not preclude senescence (age-related decrease in individual fitness) from occurring in natural populations (Nussey et al., 2012). Senescence can develop because some genes have non-separable, but typically different or opposite, functions in reproductive-age and in old individuals (antagonistic pleiotropy; Williams, 1957). Such genes, selected according to their “youthful” function, may thus impose a distinct senescent phenotype in old age. In general, however, unless a controversial formulation of group selection (Nowak et al., 2010; Wilson, 2012) is invoked, traits that would become manifest only in old age cannot evolve. This precludes the evolutionary emergence of aging programs, which have been sometimes postulated to exist (Goldsmith, 2012; Mitteldorf, 2012) in analogy to developmental and other biological programs. (By the same token, selective pressure that diminishes with age would also prevent extreme longevity from evolving, if “extreme” denotes a potential life span much longer than that imposed by extrinsic mortality in a given environment.) This and other arguments against the existence of an aging program have been discussed previously (e.g., Zimniak, 2008; Kirkwood and Melov, 2011).

The evolutionary perspective sketched out above does not specify the mechanisms that underlie aging, but it helps to narrow down the possibilities. As already discussed, an evolved deterministic aging program can be ruled out, perhaps with the exception of specific niche situations. In the absence of adaptive life-curtailing processes driven by a putative aging program, we are left with untargeted pro-aging, destabilizing phenomena which, in principle, may range from purely stochastic to side-effects of “legitimate” biochemical pathways. These destabilizing forces are counteracted by evolved, and genetically controlled, longevity assurance (or repair/maintenance) processes. The interplay of these countervailing forces determines the life span. While I have previously presented my detailed interpretation of this model (Zimniak, 2008, 2011), its central tenets bear repeating: (a) the destabilizing processes that drive aging are neither evolved nor adaptive; (b) in contrast, longevity assurance mechanisms are under genetic control; (c) together, these two opposing forces determine life span; (d) the average life span of a species is set by evolving longevity assurance mechanisms so as to optimize reproductive success under environmental conditions typical for that species.

It is important to stress that the above model allows for longevity assurance, and thus life span, being acutely regulated at the level of an organism via sensory pathways such as insulin or mTOR signaling, as long as the resulting life expectancy optimizes reproductive success under particular environmental conditions. In other words, reproductively optimal life spans evolved for different environmental situations via adaptive selection of distinct set points of anti-aging repair and/or maintenance processes. Thus, the model is fully consistent with the disposable soma theory (Kirkwood, 2005 and references therein).

What exactly, in molecular terms, are the maintenance mechanisms able to extend life? In addition to its intellectual interest, this question has considerable practical ramifications, including the holy grail of prolonging human life. A good way to approach this question is to identify first the life-curtailing destabilizing factors that are the proximal cause of aging. A focus on destabilizing factors does not imply that longevity assurance is somehow less important. As already discussed, both parts of the equation are equally significant in determining life span. However, longevity assurance mechanisms evolved in response to destabilizing factors, so defining the latter is a good point to start.

Destabilization is often thought of as a purely physical or chemical phenomenon, epitomized by the infamous (because of its incompleteness) comparison of an aging organism to a rusting car. Most emphatically, a biological system is subject to all laws of physics and will deteriorate just as a car, but this is only one of several processes relevant to a living organism. This, however, is a topic for another discussion. In the context of the present article it is important to note that destabilizing factors include, in addition to physico-chemical, also biological processes. These processes did not evolve to drive aging, at least in general. However, some side-effects of otherwise homeostatic biological reactions clearly contribute to aging (Zimniak, 2011).

Historically, the first types of reactions proposed to destabilize biological systems and to cause aging were free radical and oxidative processes (Pearl, 1928; Harman, 1956). As a consequence, even in today's literature, molecular damage is often assumed to be limited to oxidative damage, and the two terms are used interchangeably. This is unfortunate because a wide variety of errors, on scales ranging from molecular through microscopic to macroscopic, is likely to be relevant to aging.

In addition to the already mentioned oxidative and free radical damage, possible destabilizing factors are thought to include entropy-driven loss of organization inevitable in any system that is far from thermodynamic equilibrium, stochastic events inherent in biological processes which often involve relatively small numbers of molecules, modifications of essential macromolecules by reactive xenobiotics as well as by intermediary metabolites, including electrophiles derived mostly from lipid peroxidation, and protein misfolding and aggregation. Because of space limitations, I must refer the reader to my previous reviews of these topics (Zimniak, 2008, 2011) for additional details and references, as well as for a discussion of longevity assurance mechanisms able to offset the various types of damage. Here, I would like to focus on a new and radical development in the aging field, namely an attempt to falsify the above model of aging and to replace it by a new paradigm.

In a series of papers (e.g., Blagosklonny, 2006, 2007a,b, 2008, 2009, 2010a,b, 2011a,b, 2012; Blagosklonny and Hall, 2009), Mikhail Blagosklonny proposed that a novel conceptual framework is necessary to understand aging. According to the new theory, which is gaining acceptance of leading researchers in the field (Gems and de la Guardia, 2012), aging is driven not by untargeted molecular damage, but by hyperfunction and hypertrophy secondary to an inappropriate continuation into adulthood of developmental programs, in particular mTOR signaling. In this theory, mTOR, which is adaptive during growth, would become a quasi-program with detrimental consequences during adulthood, turning the model into an example of antagonistic pleiotropy (Blagosklonny, 2010b). The failure to terminate the quasi-program in adulthood could be attributed to the impossibility of evolving an off-switch in the face of a selective pressure that diminishes with age. It should be noted that, independently of hyperfunction, accumulation of molecular damage would still occur, as required by laws of physics and chemistry, but such damage would be irrelevant to aging because death triggered by hyperfunction-related pathologies would precede any life-curtailing effects of molecular damage (Blagosklonny, 2012, and other works by this author). A schematic depiction of the hyperfunction theory is shown in Figure 1A, in comparison with the molecular damage theory of aging (Figure 1B). A hypertrophy-based hypothesis has been also proposed to explain the replicative life span of yeast (Bilinski and Bartosz, 2006; Bilinski et al., 2012).

**Figure 1:**
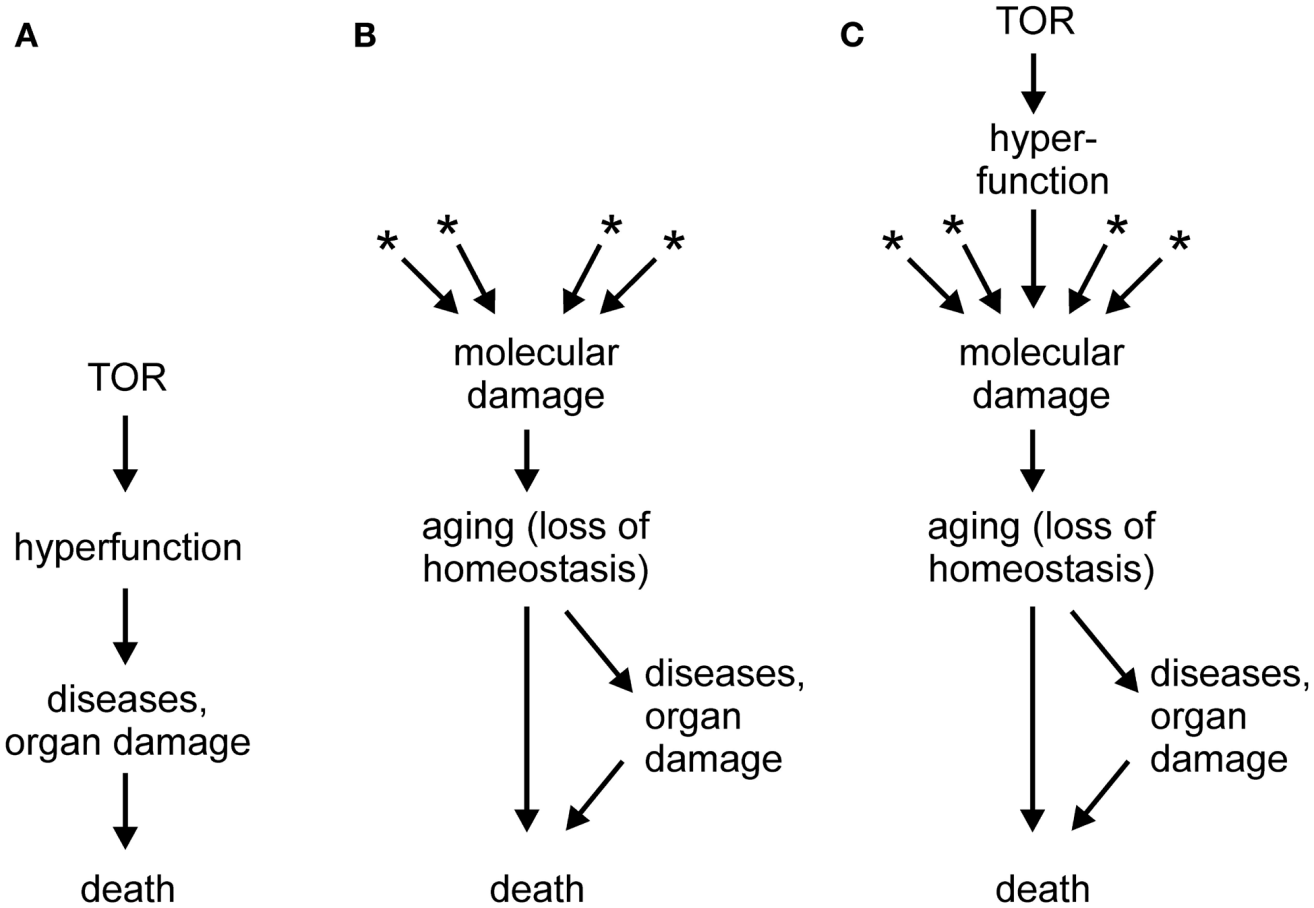
**(A)** Scheme of the hyperactivity theory of aging (based on Blagosklonny, 2011a; Gems and de la Guardia, 2012). **(B)** Scheme of the molecular damage accumulation theory of aging; asterisks denote multiple sources of molecular damage, such as electrophilic stress, oxidative stress, protein misfolding, stochastic events, and others. **(C)** Scheme of a generalized molecular damage accumulation theory of aging which includes hyperfunction/hypertrophy as a source of destabilizing molecular damage which acts in addition to other sources of damage. See text for more details.

The new perspective provided by the hyperfunction theory of aging is attractive because of recently identified deficiencies of more conventional models, especially of the oxidative stress theory. Specifically, it has been pointed out that the expected correlation between antioxidant status and longevity is not consistently observed in many experimental settings (e.g., Gems and Doonan, 2009; Perez et al., 2009; Pun et al., 2009). This may merely reflect the need for a more nuanced understanding of the chemistry and biology of oxidative stress (Gutteridge and Halliwell, 2010; Murphy et al., 2011; Halliwell, 2012). In addition, the oxidative damage theory may require modifications or refinement. For example, it has been proposed that oxidative damage may limit life span in the wild but not under protected laboratory conditions, or that oxidative stress is relevant to health span but not to life span (Salmon et al., 2010). The hyperfunction theory sidesteps these questions by declaring all molecular damage to be irrelevant to aging. The claim of hyperfunction to exclusivity is, however, worrisome for several reasons, elaborated below.

As illustrated in Figure 1A, the hyperfunction model postulates a causal chain leading from hypertrophy to macroscopic pathologies (organ damage) and to death (Blagosklonny, 2012). However, I would hesitate to accept that catastrophic events, such as a stroke in a middle-aged person or sepsis in an otherwise healthy individual, are aging. Rather, loss of homeostasis, i.e., aging, can lower cell/tissue robustness and precipitate catastrophic events (Figure 1B). If so, the hyperfunction model may be better at explaining mortality than aging. This may be considered an artificial distinction; however, it would be difficult to identify catastrophic death events in, for example, bacteria, organisms that also age (Rang et al., 2011).

Another criticism of the hyperfunction model may appear trivial. It has been claimed that atrophy, a classical sign of aging-related decline, can be in fact secondary to an initial hyperfunction and hypertrophy (Blagosklonny, 2012). At the risk of sounding petty, I would counter that with a sufficiently broad definition, almost any abnormality could be subsumed under the term hyperfunction. In fact, increased ROS production is an example of hyperfunction. However, the problem goes beyond semantics and touches on mechanism. According to the paper quoted above (Blagosklonny, 2012), hyperfunction results in hypertrophy and, eventually, in cell failure or death, i.e., atrophy. But, what is the mechanism of this chain of events? Cells and organisms are ultimately chemical systems; therefore, they are susceptible to chemical (or physical) interference. In itself, a mere increase in the abundance of an overproduced component should not matter. However, if that component interacts with normal cell constituents and interferes with their function, it causes damage – molecular damage – which may kill the cell. For example, an overproduced ligand may over stimulate or desensitize a receptor, and an overabundant protein may aggregate and interfere with intracellular trafficking, or co-precipitate with and thus withdraw essential cell constituents. Macroscopic hypertrophy can have molecular sequelae as well; for example, obesity results in a pro-inflammatory and pro-oxidant state (Grimsrud et al., 2007; Holguin and Fitzpatrick, 2010). From this point of view, hyperfunction is one of several sources of molecular damage, on equal footing with reactive metabolites, toxicants, ROS, electrophiles, stochastic events, and many others (Figure 1C).

Whereas aging is likely to have multiple contributing causes (Zimniak, 2008; Gladyshev, 2012), one of the looming questions in gerontology is whether any one type of damage predominates, and if so, which. This question is as important as it is difficult to answer, in part because many seemingly distinct experimental interventions lead to overlapping or identical molecular perturbations of a biological system. Among the contenders, oxidative damage has lost much of its appeal, perhaps prematurely, whereas protein misfolding/aggregation is gaining support (Morimoto and Cuervo, 2009; Morimoto et al., 2011). Even if hyperfunction turns out to be the predominant driver of aging, I propose that it does so by causing molecular damage, rather than by killing organisms through triggering catastrophic organ failures. Thus, regardless of its nature, molecular damage remains the proximal cause of aging (Figure 1C).

The advent of the hyperfunction theory of aging has been compared to the replacement of the geocentric with the heliocentric worldview (Gems and de la Guardia, 2012). Within this rather grand conceptual framework, I may be seen as an old-timer who desperately tries to salvage a doomed theory by piling up epicycles. Perhaps so – time will tell. Meanwhile, I would like to invoke another old-timer, William of Ockham. Wielding his razor, I propose that, if hyperfunction is treated as a destabilizing process that generates molecular damage, all experimental evidence can be accommodated by the generalized molecular damage theory of aging, without the need to establish a new paradigm.

## References

[B1] Bilinski T. Bartosz G. (2006). Hypothesis: cell volume limits cell divisions. Acta Biochim. Pol. 53, 833–835 17106512

[B2] Bilinski T. Zadrag-Tecza R. Bartosz G. (2012). Hypertrophy hypothesis as an alternative explanation of the phenomenon of replicative aging of yeast. FEMS Yeast Res. 12, 97–101 10.1111/j.1567-1364.2011.00759.x 22093953

[B3] Blagosklonny M. V. (2006). Aging and immortality: quasi-programmed senescence and its pharmacologic inhibition. Cell Cycle 5, 2087–2102 10.4161/cc.5.14.3113 17012837

[B4] Blagosklonny M. V. (2007a). Paradoxes of aging. Cell Cycle 6, 2997–3003 10.4161/cc.6.1.3682 18156807

[B5] Blagosklonny M. V. (2007b). Program-like aging and mitochondria: instead of random damage by free radicals. J. Cell. Biochem. 102, 1389–1399 10.1002/jcb.21602 17975792

[B6] Blagosklonny M. V. (2008). Aging: ROS or TOR. Cell Cycle 7, 3344–3354 10.4161/cc.7.17.6626 18971624

[B7] Blagosklonny M. V. (2009). TOR-driven aging: speeding car without brakes. Cell Cycle 8, 4055–4059 10.4161/cc.8.24.10310 19923900

[B8] Blagosklonny M. V. (2010a). Rapamycin and quasi-programmed aging: four years later. Cell Cycle 9, 1859–1862 10.4161/cc.9.4.10766 20436272

[B9] Blagosklonny M. V. (2010b). Revisiting the antagonistic pleiotropy theory of aging: TOR-driven program and quasi-program. Cell Cycle 9, 3151–3156 10.4161/cc.9.4.10766 20724817

[B10] Blagosklonny M. V. (2011a). Hormesis does not make sense except in the light of TOR-driven aging. Aging (Albany NY) 3, 1051–1062 22166724 10.18632/aging.100411 PMC3249451

[B11] Blagosklonny M. V. (2011b). Molecular damage in cancer: an argument for mTOR-driven aging. Aging (Albany NY) 3, 1130–1141 22246147 10.18632/aging.100422 PMC3273893

[B12] Blagosklonny M. V. (2012). Cell cycle arrest is not yet senescence, which is not just cell cycle arrest: terminology for TOR-driven aging. Aging (Albany NY) 4, 159–165 22394614 10.18632/aging.100443 PMC3348476

[B13] Blagosklonny M. V. Hall M. N. (2009). Growth and aging: a common molecular mechanism. Aging (Albany NY) 1, 357–362 20157523 10.18632/aging.100040 PMC2806018

[B14] Comfort A. (1956). The Biology of Senescence. New York: Rinehart & Co

[B15] Gems D. de la Guardia Y. (2012). Alternative perspectives of aging in *C. elegans*: reactive oxygen species or hyperfunction? Antioxid. Redox Signal. (in press). 10.1089/ars.2012.4840 PMC5395017 22870907

[B16] Gems D. Doonan R. (2009). Antioxidant defense and aging in *C. elegans*: is the oxidative damage theory of aging wrong? Cell Cycle 8, 1077–1083 10.4161/cc.8.11.8595 19411855

[B17] Gladyshev V. N. (2012). On the cause of aging and control of lifespan: heterogeneity leads to inevitable damage accumulation, causing aging; control of damage composition and rate of accumulation define lifespan. Bioessays (in press). 10.1002/bies.201200092 PMC3804916 22915358

[B18] Goldsmith T. C. (2012). On the programmed/non-programmed aging controversy. Biochemistry Mosc. 77, 729–732 10.1134/S000629791207005X 22817536

[B19] Grimsrud P. A. Picklo M. J. Sr. Griffin T. J. Bernlohr D. A. (2007). Carbonylation of adipose proteins in obesity and insulin resistance: identification of adipocyte fatty acid-binding protein as a cellular target of 4-hydroxynonenal. Mol. Cell. Proteomics 6, 624–637 10.1074/mcp.M600120-MCP200 17205980

[B20] Gutteridge J. M. Halliwell B. (2010). Antioxidants: molecules, medicines, and myths. Biochem. Biophys. Res. Commun. 393, 561–564 10.1016/j.bbrc.2010.02.071 20171167

[B21] Halliwell B. (2012). Free radicals and antioxidants: updating a personal view. Nutr. Rev. 70, 257–265 10.1111/j.1753-4887.2012.00476.x 22537212

[B22] Harman D. (1956). Aging: a theory based on free radical and radiation chemistry. J. Gerontol. 11, 298–300 13332224 10.1093/geronj/11.3.298

[B23] Holguin F. Fitzpatrick A. (2010). Obesity, asthma, and oxidative stress. J. Appl. Physiol. 108, 754–759 10.1152/japplphysiol.00702.2009 19926826

[B24] Kirkwood T. B. (2005). Understanding the odd science of aging. Cell 120, 437–447 10.1016/j.cell.2005.01.027 15734677

[B25] Kirkwood T. B. Melov S. (2011). On the programmed/non-programmed nature of ageing within the life history. Curr. Biol. 21, R701–R707 10.1016/j.cub.2011.07.020 21959160

[B26] Medawar P. B. (1952). An Unsolved Problem of Biology. London: H. K. Lewis

[B27] Mitteldorf J. J. (2012). Adaptive aging in the context of evolutionary theory. Biochemistry Mosc. 77, 716–725 10.1134/S0006297912070036 22817534

[B28] Morimoto R. I. Cuervo A. M. (2009). Protein homeostasis and aging: taking care of proteins from the cradle to the grave. J. Gerontol. A Biol. Sci. Med. Sci. 64, 167–170 10.1093/gerona/gln071 19228787 PMC2655025

[B29] Morimoto R. I. Driessen A. J. M. Hegde R. S. Langer T. (2011). The life of proteins: the good, the mostly good and the ugly. Nat. Struct. Mol. Biol. 18, 1–4 10.1038/nsmb0111-1 21209622

[B30] Murphy M. P. Holmgren A. Larsson N.-G. Halliwell B. Chang C. J. Kalyanaraman B. Rhee S. G. Thornalley P. J. Partridge L. Gems D. Nystrom T. Belousov V. Schumacker P. T. Winterbourn C. C. (2011). Unraveling the biological roles of reactive oxygen species. Cell Metab. 13, 361–366 10.1016/j.cmet.2011.03.010 21459321 PMC4445605

[B31] Nowak M. A. Tarnita C. E. Wilson E. O. (2010). The evolution of eusociality. Nature 466, 1057–1062 10.1038/nature09205 20740005 PMC3279739

[B32] Nussey D. H. Froy H. Lemaitre J. F. Gaillard J. M. Austad S. N. (2012). Senescence in natural populations of animals: widespread evidence and its implications for bio-gerontology. Ageing Res. Rev. (in press). 10.1016/j.arr.2012.07.004 PMC4246505 22884974

[B33] Pearl R. (1928). The Rate of Living. London: University of London Press

[B34] Perez V. I. Bokov A. Van Remmen H. Mele J. Ran Q. Ikeno Y. Richardson A. (2009). Is the oxidative stress theory of aging dead? Biochim. Biophys. Acta 1790, 1005–1014 10.1016/j.bbagen.2009.06.003 19524016 PMC2789432

[B35] Pun P. B. L. Gruber J. Tang S. Y. Schaffer S. Ong R. L. S. Fong S. Ng L. F. Cheah I. Halliwell B. (2009). Ageing in nematodes: do antioxidants extend lifespan in *Caenorhabditis elegans*? Biogerontology 11, 17–30 10.1007/s10522-009-9223-5 19350411

[B36] Rang C. U. Peng A. Y. Chao L. (2011). Temporal dynamics of bacterial aging and rejuvenation. Curr. Biol. 21, 1813–1816 10.1016/j.cub.2011.09.018 22036179

[B37] Salmon A. B. Richardson A. Perez V. I. (2010). Update on the oxidative stress theory of aging: does oxidative stress play a role in aging or healthy aging? Free Radic. Biol. Med. 48, 642–655 10.1016/j.freeradbiomed.2009.12.015 20036736 PMC2819595

[B38] Williams G. C. (1957). Pleiotropy, natural selection, and the evolution of senescence. Evolution 11, 398–411 10.2307/2406060

[B39] Wilson E. O. (2012). The Social Conquest of Earth. New York: Liveright Publishing Co./W. W. Norton

[B40] Zimniak P. (2008). Detoxification reactions: relevance to aging. Ageing Res. Rev. 7, 281–300 10.1016/j.arr.2008.04.001 18547875 PMC2671233

[B41] Zimniak P. (2011). Relationship of electrophilic stress to aging. Free Radic. Biol. Med. 51, 1087–1105 10.1016/j.freeradbiomed.2011.05.039 21708248 PMC3156362

